# “After Cutting It, Things Have Never Remained the Same”: A Qualitative Study of the Perspectives of Amputees and Their Caregivers

**DOI:** 10.1111/hex.70148

**Published:** 2025-01-09

**Authors:** Esther Ohenewa, Joana Salifu Yendork, Benjamin Amponsah, Frances Emily Owusu‐Ansah

**Affiliations:** ^1^ Department of Behavioural Sciences Kwame Nkrumah University of Science and Technology Kumasi Ghana; ^2^ Psychiatric Unit, Komfo Anokye Teaching Hospital (KATH) Kumasi Ghana; ^3^ Department of Psychology University of Ghana Accra Ghana

**Keywords:** amputation, amputees, caregivers, coping, well‐being

## Abstract

**Introduction:**

Physical and emotional loss from amputation and associated physical disability are associated with adverse physical and psychological experiences. However, little research, within the Ghanaian context, has focused on the impact of amputation on the well‐being of amputees and their caregivers and the coping strategies they use to mitigate challenges experienced. Therefore, the present study explored the impact of amputation on the well‐being of amputees and caregivers, and the coping strategies they employ to manage distress associated with amputation and caregiving.

**Methods:**

The aim of this present study was explored using a qualitative descriptive design. Twenty (20) participants (comprising 10 amputees and 10 caregivers) responded to a semi‐structured interview guide.

**Results:**

Findings show that whereas amputees experienced phantom limb sensation and loss of job, caregivers reported economic hardship and fear. Though stress was a common experience, the source of stress was different for amputees and caregivers. Regarding coping, whereas amputees used social distancing, social reference and social support to cope with their traumatic ordeal, caregivers simply coped by encouraging themselves. Both amputees and caregivers used religious coping.

**Conclusion:**

Amputees and their caregivers experience varied stressors yet whereas the amputees get the needed support to deal with their predicament, caregivers lack adequate support.

**Patient or Public Contribution:**

The findings underscore the need for diverse support systems and psychoeducation on adaptive coping strategies for amputees and caregivers.

## 1 Introduction

Loss can manifest in various forms, including the death of a loved one, disability, or failure in personal or professional spheres [1]. One significant form of loss is amputation, which causes both physical and emotional pain, often leading to complex psychological experiences for the affected individuals [2, 3, 4, 5, 6, 7, 8]. Yuan et al. [9] reported that globally, the incidence and prevalence of traumatic amputation as of 2019 was 13.23 million with the number increasing gradually. Other conditions that may result into amputation include chronic conditions such as diabetes and peripheral artery disease [10, 11]. The impact of amputation can vary greatly depending on the cause. Traumatic amputations, often resulting from accidents or violence, can trigger sudden psychological responses such as shock, posttraumatic stress disorder (PTSD), and difficulties in adjusting to the loss [12]. In contrast, amputations due to medical conditions like diabetes or vascular disease are typically associated with long‐term health challenges, where individuals may experience a sense of loss, frustration, or relief if the procedure prevents further health complications [13]. Congenital amputations, where individuals are born without a limb, present unique challenges related to self‐image and societal acceptance, although the emotional impact tends to be more gradual [14]. For amputations caused by diseases such as cancer or infections, the emotional distress is often compounded by the uncertainty surrounding the underlying illness and grief over body loss [15].

Amputees employ a range of coping strategies to manage distress, including both adaptive and maladaptive methods that may affect their well‐being [5, 16]. Positive coping strategies have been linked to better psychological adjustment, and promoting these can facilitate emotional recovery [17]. Despite the attention given to amputees' emotional experiences, the emotional impact on caregivers—who provide essential physical and emotional support during recovery—has been less thoroughly explored yet both amputees and their caregivers often experience a range of negative emotions following amputation [18]. Amputees face a range of challenges, including mobility restrictions, phantom limb sensations, and the need for prosthetic devices [3, 4, 7, 8, 19, 20], all of which necessitate ongoing care and adjustment. Caregivers often face significant emotional strain, physical exhaustion, and financial burdens, which can affect their mental health and well‐being [21]. The mutual dependence between amputees and caregivers plays a crucial role in rehabilitation, and without proper support, caregivers may experience significant mental health challenges [22, 23].

The relationship between amputees and their caregivers is marked by interdependence, which supports successful rehabilitation. However, caregiving can be overwhelming, leading to the need for comprehensive support systems to address both the amputees' and caregivers' psychological and emotional needs, as caregiver well‐being influences the well‐being of care recipient. Unfortunately, a literature search reveal that unlike the amputees, there are no initiatives or psychosocial interventions for caregivers of amputees in Ghana. This study seeks to explore the impact of amputation on the well‐being of both amputees and caregivers, focusing on the coping strategies they employ to manage potential distress associated with amputation and caregiving. This study aimed to answer the following research questions: (1) What is the impact of amputation on amputees and caregivers? (2) How do amputees and caregivers cope with the distress associated with amputation and caregiving?

## 2 Materials and Methods

### 2.1 Design

This study used a qualitative descriptive research design which encourages participants to provide factual and in‐depth responses to questions about their unique experiences [24]. Qualitative descriptive research designs offer a number of benefits such as allowing for content and descriptive analysis of data [24]. Considering that the aim of this research was to capture the impact of amputation and explore the coping strategies amputees and their caregivers used, a qualitative descriptive research design allow for an “…inductive exploration of the data to identify recurring themes, patterns, or concepts and then describing and interpreting” [25, p. 130].

Data were gathered using a semi‐structured interview, which is flexible and allows the researcher to explore other areas that may surface in the course of the interview [26]. Its flexibility and adaptability make it distinctive from a structured interview guide [26]. Interviews were audio‐taped and in certain circumstances where participants felt uncomfortable with being recorded, the contents of the interviews were manually recorded using a personal notepad and a pen. The semi‐structured interview guide contained eight (8) questions that delved into how participants felt the amputation had impacted their lives and how they dealt with the challenges they experienced. Sample question included: “What were your thoughts at the time you heard of/or experienced the situation*”?* and “How do you deal with the emotional experiences resulting from this encounter?*”*. Out of the 20 participants (comprising 10 amputees and 10 caregivers) that were engaged, only two individuals were uncomfortable being recorded. Their responses to the interview questions were manually recorded.

### 2.2 Procedure and Ethical Considerations

Ethical clearance for the study was sought and obtained from the Ethics Committee for Humanities (ECH: 016/16–17) at the University of Ghana, Legon. Afterwards, permission for data collection was granted by St. Joseph Hospital and Nsawam Orthopedic Center, both located in the Eastern Region of Ghana. The public relations officers at these institutions facilitated the introduction of the first author (E.O.) to patients and caregivers. To be included in the study, participants had to be 18 years old and above and should have been an amputee or caregiver of an amputee for not more than 6 months. Of the 29 individuals comprising 13 amputees and 16 caregivers that were purposefully sampled, 20 participants (10 amputees and 10 caregivers) consented to participate in the study. Interviews were conducted by the first author only at the St. Joseph Hospital (7 interviews) and Nsawam Orthopedic Center (13 interviews), in either Akan or English Language. Each interview lasted between 30 and 60 min and was transcribed verbatim for further analysis. At the time of data collection, the first author had a bachelor's degree in psychology and was pursuing a master's degree in clinical psychology. She was guided by the second, third and fourth authors who had a doctorate in psychology and were experienced in both quantitative and qualitative research methodologies.

### 2.3 Rigour and Trustworthiness of Results

The study followed Maxwell [27] and Guba's [28] guidelines to ensure the validity and trustworthiness of the findings. Maxwell [27] proposes an accurate representation of respondents' accounts, interpretation based on participants' perspectives, and consideration of alternative views. Data were collected without a predefined framework and analysed from the respondents' viewpoints, with verbatim transcription to enhance reliability [29].

The study adhered to Guba's [28] criteria for trustworthiness—credibility, dependability, transferability, and confirmability. Credibility was ensured through piloting of the interview guide to ensure that the questions on the interview guide truly reflect the research questions and were appropriate for the study participants. We also did member checks by engaging with some participants after data analysis to validate themes. Dependability was ensured by providing detailed research procedures and instruments [30]. Transferability was achieved through the use of purposive sampling and by ensuring diverse representation of amputees and caregivers [31]. Confirmability was maintained through the use of multiple coders and a transparent audit trail.

### 2.4 Authors Position Statement

The first, second and third authors participated in the study conceptualisation, design, data collection, analysis and interpretation of results, and review of the manuscript. The fourth author participated in the analysis and interpretation of the results, as well as review of the manuscript. All authors had no prior relationship with the research settings and participants and no experience with amputation. While every effort was made to maintain objectivity, the authors acknowledge potential biases stemming from their outsider position and lack of personal experience of amputation, which may have influenced the interpretation of findings.

### 2.5 Data Analysis

The interviews were transcribed verbatim and analysed using Braun and Clarke's [32] six‐step thematic analysis process: Familiarisation with the data, Generation of initial codes, Search for themes, Review of themes, Defining and naming themes, and Producing the final report. Thematic analysis involves identifying and analysing patterns and themes in data set [33]. After the first authors transcribed the interview, the team ensured accuracy by cross‐checking the audio recordings and revising the transcripts as needed. Then the first and second authors familiarised themselves with the data by reading through the transcripts and highlighting relevant segments that align with the research focus. Initial codes were generated independently by the first and second authors, and then reviewed and refined collectively. The codes were organised into patterns, which formed the themes. These themes were further reviewed and refined for clarity, with redundant themes combined. Each theme's essence was examined and defined, leading to a final, detailed analysis supported by relevant literature and theory.

## 3 Findings

### 3.1 Demographic Information About Study Participants

**Information About Amputees**: There were six males and four females. There was one participant in the age range of 18–23 years, two were aged between 23 and 28 years, one was 28–33 years, three were 33–38 years, one each for 38–43, 43–48 and 48–53. Regarding the types of amputation, seven had undergone lower leg amputation, one had arm amputation and two had foot amputation. Regarding employment status, three were unemployed, two were students, three were mechanics, one was a waitress, and one was a health worker.

**Information About Caregivers**: There were eight females and two males. One caregiver was aged between 18 and 23 years, three were aged between 23 and 28 years, three were between 28 and 33 years, two were between 38 and 43 years and one was aged 53 and 58 years. Regarding relations to amputees, one was a parent, one was a spouse, one was a ward, three were siblings, and four were other relatives to the amputees. Concerning the profession, two were unemployed, three were students, three were petty traders and two were health workers.

**Figure 1:**
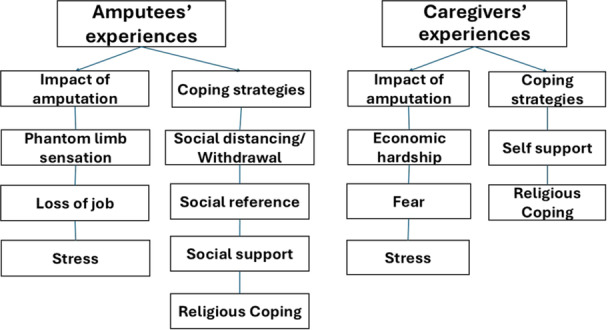
Summary of findings.

The findings of the data analyses are presented below. The experiences of amputees were compared with those of their caregivers, for a full appreciation of the discrepancies and similarities in their experiences. The themes that emerged from the data have been organised graphically in Figure 1.

## 4 The Impact of the Experience of Amputation

This theme explored how amputation impacted the well‐being of amputees and their caregivers. For amputees, the distress they experienced included phantom limb sensation and a change in vocation, whereas, for the caregivers, their experiences included fear and economic hardship. The experience of stress was reported by both amputees and their caregivers.

### 4.1 Influence of Amputation on Amputees

**Phantom Limb Sensation:** Six amputees reported experiences whereby they felt the presence of the primary organ that had been amputated. A constant feeling that one's primary organ is still present when evidently the primary organ is no more, is psychologically traumatising for the amputees. It can disrupt daily functioning because the individual will need a constant reminder that their primary organ is no more.

*… Sometimes my leg can itch for a while but when I try to scratch it, I realize that my leg is no longer there* (Amputee, Female, 49 years old, Unemployed nurse)

*The nurse explained my situation to me so I understand that they had to amputate but I still feel like my leg is there. I can get up suddenly only to realize that my leg is no more* (Amputee, Female, 24 years old, School drop‐out)

**Loss of Job:** Three amputees recounted that following the loss of their limb, they lost their jobs and had to contemplate changing vocations. For some, this resulted in a state of confusion as to from where to start life. Changing a vocation with complete unpreparedness could escalate into serious psychological distress. While some amputees were in a state of confusion, others disclosed that for them death would have been a better option. The frustrations experienced are reflected in the narratives below:

*I was thinking that, now I won't be able to …work like I used to do. I sell things by the roadside and sometimes you have to run after the cars passing by just to sell these things, how do I do that now without my leg? and also I have a wife and kids, so now I have changed my job else we [reference to himself and his wife and kids] will starve…* (Amputee, Male, 35 years old, Unemployed)

*…eheeee and with this, after cutting it things have never remained the same. Although I agreed that they should cut my leg, but it has affected my job. I had to quit my job. I am now searching for another job that I can do easily…*(Amputee, Male, 42 years old, Mechanic)

### 4.2 Influence of Amputation on Their Caregivers

**Economic Hardship** ***:*** Thematic analyses revealed that amputation led to economic hardship, which greatly impacted the lives of the caregivers of the amputees (*n* = 6). The amputee was shielded from feeling the full impact of the economic hardship because the caregivers viewed the amputee, as vulnerable and in need of proper care or food to recover from his or her ordeal. This is the denial of the self to please or care for the other. According to some caregivers:

*…things really got difficult…there were days we chewed just roasted corn and groundnuts and we gave her [amputee] proper food to eat… there were days we chewed in front of* her (Caregiver, Female, 39 years old, Aunt, Petty Trader)

*We both don't work, so taking care of ourselves while in this situation is a struggle…* (Caregiver, Female, 28 years old, Sibling, Student)

**Fear:** Caregivers revealed that they could not comprehend why this (a close relative being amputated) should happen to them (*n* = 4). This opened a new window for fear, that is the fear of the unknown. Some caregivers explained that

*…I am scared…because I have never seen something like this and it has happened to me so sometimes I get scared…* (Caregiver, Female, 24 years old, Wife, Petty Trader)

*One day he left home to attend to his duties, the next day I was called that his leg had been amputated, how!…At first, I was scared to go and see him at the hospital. This thing [reference to the amputation] can happen to anyone at all, even me…this world is scary* (Caregiver, Male, 42 years old, Relative, Unemployed)

### 4.3 Amputees and Caregivers Experience of Stress

Both amputees and their caregivers reported stressful encounters although the source of the stress was different. For the amputees, their stress resulted from adapting to prosthetic legs whereas for the caregivers, their stress resulted from the caregiving activities to their relatives following their amputation.

Eight amputees narrated stressful incidences brought upon them due to amputations. Their stress starts right from the time they wake up and must wear an artificial body part. They narrated the physical pains they had to endure and the consequent limitations on even choice of outfit. This is an adjustment and adaptation phase that can be stressful for the individual without the right kind of support. Due to this stress, some preferred to stay in their rooms rather than go through the stress that prosthetic legs present to them:

*Sometimes when I wake up, I just assume that I could just get up from bed and start moving but I need to take my leg [prosthetic leg] and wear it. After wearing it, I feel pains on taking the first few steps…every morning when I get up and wear it, I feel pain upon using it to take steps before it becomes normal. Sometimes too, it feels as though it wants to fall off… when you wear skirt like this… it can remove … you don't feel [comfortable]… even when you are going to bath you have to take it along to the bathroom and when you are bathing you need remove it* (Amputee, Female, 26 years old, Unemployed nurse)

*It is so much work having to wake up in the morning and put on your fake leg before going out. I just prefer to stay in my room rather than go through the process of wearing it* (Amputee, Male, 35 years old, Unemployed)

Likewise, 10 caregivers described the stress they go through caring for the needs of their relative. Some described their daily routine of providing the needs of the amputee. This is a shift from one's way of life to make life comfortable for another individual. The adjustment process was equally stressful for the caregivers as some had to abandon their work to stay beside the amputee and provide support:

*… I stayed with him here because there was no one who could come and stay here…I had to gather courage to come and stay here. I sleep here…I get up early to clean him up and cook for him…I stay by his side so I help him with things he needs until it is time for us to leave them…I have not been to work ever since he came here…* (Caregiver, Female, 24 years old, Wife, Petty Trader)

*I do everything for him and take care of myself too…his parents have passed away so you can just imagine!…It is not easy* (Caregiver, Male, 42 years old, Relative, Unemployed)

## 5 Coping Strategies for Managing Distresses

The coping mechanisms adopted by both amputees and their caregivers following amputation were explored in this study. Findings show that whereas amputees used social distancing, social reference and social support as a means of coping with their traumatic ordeal, caregivers just encouraged themselves as a means of dealing with theirs. However, a common means of dealing with their experiences was religious coping through their belief in the power of an extraordinary being and prayers.

### 5.1 Coping Strategies Unique to Amputees

**Social Distancing/Withdrawal:** Five amputees felt the need to create a gap between themselves and society. Amputees disclosed that though creating a gap between themselves and society made them unhappy, they still felt the need to do it. They just did not want to go anywhere. This was a different way of life compared to their premorbid way of life. It was quite unfulfilling for the amputees and some narrated how unhappy they were about their situation:

*… when I was at home… I could go out… but since I went back, I am unable to at all… for you to see me outside even is difficult… I don't want to go anywhere…so personally I wasn't really that happy (*Amputee, Male, 35 years old, Unemployed)

*I don't go out anymore. What am I going to do outside?… People just keep looking at me when I pass by…I am not happy about it but I also don't like the way people keep looking at me when I go out so I will rather stay indoors* (Amputee, Female, 30 years old, Waitress)

**Social Reference:** Four amputees reported that comparing their situation to others assuaged the gravity of their ordeal and lessened their emotional experience. The comparison brought within the amputee some gratitude as they came to the realisation that their predicament was better compared with others:

*…when I see other people, some with both legs cut off, some have theirs cut at other places, then my own … becomes small* (Amputee, Male, 42 years old, Mechanic)

*…even though I don't think I deserve what am going through, other people go through worse situation than mine. Some die and I am alive…* (Amputee, Female, 49 years old, Nurse)

**Social Support:** Ten amputees reported relying on families and significant others for strength to deal with their difficult experiences. Thus, although the amputees acknowledged their limitations now that they are handicapped, the love and support of significant others in their lives encouraged them to give their best in life regardless of their situation. They were grateful for the support they receive

*… if you are going through such a situation and you don't get the love of your family or something like that, it may cause you to think about it a lot and you may also end up dying… I sometimes cry over the fact that now that I had completed school, that I'm supposed to be working and this has happened to me… but I get advice from my parents and my church members also come around to advise me. That is what has encouraged me to continue [living]* (Amputee, Female, 26 years old, Unemployed nurse)

*I don't know what I would have done without the support of my family. They have really helped me…* (Amputee, Male, 35 years old, Unemployed)

### 5.2 Coping Strategy Unique to Caregivers

**Self‐Support:** Eight caregivers reported that they had to encourage themselves. Caregivers felt the need to draw closer to the amputees and be emotionally strong as they undertake their caregiving responsibilities. In the process, they shield their (caregivers) emotional pain and prevent themselves from expressing negative emotions when they were around the amputee:

*…when it happens that way… you just have to encourage yourself…and focus on helping the person who needs help the most* (Caregiver, Female, 39 years old, Aunt, Petty Trader)

*…in situations like this no one can help you…you have to be strong. I can't be affected by the situation else how will this boy survive?* (Caregiver, Male, 42 years old, Relative, Unemployed)

### 5.3 Religious Coping Among Amputees and Their Caregivers

Seven amputees reported the use of prayers and belief in God as a coping strategy to deal with their experience. In some cases, it was people in the larger community who encouraged the amputees to rely on God. Some amputees were still in their dark shells trying to find meaning to their situation when their attention was redirected to their object of worship. Some revealed how they were encouraged to surrender their problems to God and implore Him to make their lives better:

*They told me to leave everything to God and that it's more important to have life and strength…so I should leave all to God and just pray that things will get better so I listened* (Amputee, Female, 30 years old, Waitress)

*…That day [reference to the day she was told they had to amputate her leg], I really prayed and then my brother encouraged me and told me that in anything, he will support me. And he supported me in prayers* (Amputee, Female, 26 years old, Unemployed nurse)

Similar to amputees, eight caregivers depended on their object of worship as well. The interesting difference was that while amputees depended on their object of worship because people in the community encouraged them to do so, caregivers naturally, turned to their object of worship for support and provision of their vital needs. According to a caregiver:

*God says in every situation we should call on him and He will answer. It gets to a point that I tell God we have nothing to even eat, or to even give to the sick person, but before the next day God is able to provide…* (Caregiver, Female, 39 years, Aunt, Petty Trader)

The reliance on a Supreme Being also made some caregivers hopeful.

*[when I think of it] the only thing I say is God is alive once he did not die and I am also not dead God will bless us in the future. Maybe, help will come from somewhere so that is what I use to encourage myself …that God will lift his hands in my life* (Caregiver, Female, 24 years old, Wife, Petty Trader)

## 6 Discussion

This study explored the distress associated with amputation and the coping strategies of both amputees and their caregivers. With regard to distress, findings indicate that whereas amputees uniquely experience phantom limb sensation and loss of job, caregivers uniquely experience economic hardship and fear. Both amputees and caregivers experience stress but the sources of the stress are different. Amputees' stress resulted from adaptation to prosthetic legs, and self‐alienation whereas the caregivers' stress resulted from the ‘burden’ of caregiving activities. Concerning coping strategies, amputees uniquely used social distancing, social reference and social support but caregivers just encouraged themselves, while relying on their religious beliefs. However, both amputees and caregivers use religious coping through their belief in the power of an extraordinary being and prayers.

Phantom limb sensation (PLS), which is the feeling of the presence of a lost body part, is a common experience following amputation [34, 35]. Losing one's body part is in itself traumatic, and the constant feeling that the lost body part is still present only further heightens one's state of denial and significantly impacts one's mental health. The experience of phantom limb sensation and loss of job among amputees in this present study is consistent with previous research [7, 8]. Abouammoh et al. [36] reported that physical adjustment contributed to amputees' quality of life as it places some form of restrictions on one's functional ability. Thus, if the amputee's vocation cannot accommodate these restrictions it may result in a change of vocation. The psychological implication of this is that the amputee may have to settle for a less satisfying job because of the disability.

Caregivers' experience of economic hardship supports the findings of Ae‐Ngibise et al. [37] and Volker [38] who reported that caregivers experienced various degrees of financial burden resulting from taking care of the needs of their relatives who may also be experiencing financial problems resulting from the disability from amputation. Generally, amputees experience some level of financial difficulty because of high medical costs and job loss [39, 40]. Further, any form of amputation restricts the individual in the type of jobs they can engage. Thus, amputees only look for jobs that can accommodate their deficits and provide them with financial assistance, yet most of these jobs are low‐paying. Given amputees' financial constraint due to their handicap, caregivers often go beyond providing psychological and physical care to the extent of supporting amputees financially which only places a strain on their finances [41]. The experience of fear in the caregivers resulted from their shared experience of trauma associated with amputation which tends to trigger traumatic stress in caregivers [42, 43]. Traumatic stress in turn is associated with increased anxiety [44]. This traumatic experience is usually not anticipated and the new realisation of the relative's ordeal and the burden they have to bear creates fear and panic, symptomatic of anxiety.

Our finding that amputees were stressed from having to go through the daily routine of adjusting to their prosthetic devices and make use of it despite the discomfort‐ is corroborated by previous works that evidence the burden of prosthesis adjustment [41]. The use of the prosthesis has implications for body image and mental health because getting used to a technological device to assume the role of primary organ does not come with ease. Therefore, it is not surprising that amputees report body image distress and associated posttraumatic stress disorder [8, 36].

Unlike the amputees, caregivers' stress was associated with caregiving role. There is much evidence to suggest that the burden of care among caregivers of amputees is high [41, 45, 46, 47, 48]. This is because caregivers make major life adjustments such as changing daily routine and prior roles, taking on new responsibilities, and/or cancelling plans to accommodate their new role with little or no social support [45, 48].

The use of social distancing, social reference and social support by amputees is at variance with the findings of Garafalo [49] and Kashani et al. [50] who reported that amputees make use of excessive alcohol consumption and drugs as a means of dealing with their distress. However, it was not surprising that amputees avoided larger communal interactions after amputation. Charlton and Thompson [51] explain that when an event is carefully placed in the past, distancing is more easily adopted. When the cause of one's amputation is as a result of a traumatic event and one is focusing on healing from such experience, a typical response will be to avoid communications surrounding that experience. Thus, it can be argued this is more likely to be achieved when one engages less with people of the larger community and potential reminders. Another possible reason could be that the amputee wants to avoid the pity glances from the larger community. Further, some amputees compared their situation to other people whose conditions were worse than theirs, and that made them feel better about their condition. This is a similar concept in downward comparison, which is a coping mechanism used to reduce the psychological impact of negative life events [52]. In downward comparison, one arranges comparison in such a way that the outcome of the comparison is more favourable to the self than the other [52]. Regardless of the seemingly negative connotative of this coping mechanism, it presents a dimension of hope to the self or the individual. It gives one hope that if the ‘other’ whose situation appears to be worse than the ‘self’ is surviving, then life is worth living and the ‘self’ could find reasons not to give up. Evidence suggests that amputees make use of social support as a way of dealing with their ordeal [16, 53]. In this study, amputees relied greatly on the love and care of relatives, loved ones and friends for support in dealing with their ordeal. Thus, social support is a form of collective coping mechanism, which affords an individual the opportunity to solicit for comfort from members of their in‐group in distressing events [54]. This finding reinforces the idea that Africans are collective in nature [55].

The use of self‐support by caregivers, whereby caregivers relied on no one but themselves for strength, is an interesting finding, that supports Ae‐Ngibise et al.'s (2015) and Costa et al.'s (2020) finding on lack of social support for caregivers. Despite the emotional stressors they must deal with, their only form of support was religious support and prayers [56]. Caregivers do not make use of collective coping perhaps because they feel that cannot rely on anyone but themselves to be strong for the amputee. Literature has established that in the case of amputation, attention is concentrated on the amputees to the neglect of the caregivers [38, 57, 58]. Thus, this finding shows that caregivers are neglected to deal with their distress following amputation.

Religious coping, in the form of belief in the power of God and prayers, was used by both caregivers and amputees in this present study. This finding corroborates the results of Ae‐Ngibise et al.'s (2015) and Livneh et al.'s (2000) study that reported that amputees and caregivers use spirituality and religious prayers as a means of coping with their ordeal. Further, this finding is consistent with evidence of studies that suggest that Ghanaians are highly religious [59] and are more inclined to use religious coping when faced with distress [60, 61, 62, 63]. Religious coping provides them with the courage to face their predicament and increases their faith in God as one who cures and heals diseases in the body [60]. Thus, in an uncontrollable situation such as amputation where there seem to be limitations in human capabilities, religious coping seems ideal.

### 6.1 Limitations of Study

While the present study has highlighted the nature of distress and coping strategies used by Ghanaian amputees and caregivers, some limitations need to be acknowledged. First, we used a small sample of amputees and caregivers. Hence, the findings cannot be generalised to the population of amputees and caregivers in Ghana. Future research should consider using a larger sample i.e. nationally representative to document broader experiences among the population of Ghanaian amputees and caregivers. Second, the sample was taken from the hospitals, so the experiences shared may not reflect those of amputees within the community. Future studies should consider sampling from both the hospitals and within the communities in Ghana. This will ensure that diverse experiences are brought to light to inform broader interventions that will enhance the well‐being of amputees and caregivers.

### 6.2 Implications for Research and Practice

Given that amputees and caregivers experienced varied stressors, both groups will benefit from psychoeducation on stress management techniques that can be delivered when amputees and their caregivers visit the hospitals for review, thus reaching as many amputees and their caregivers as possible. Also, the findings of this study show that caregivers lack adequate support and will benefit from both formal and informal support systems. Formally, the Government of Ghana can consider providing financial support to caregivers to ease their burden. Informally, families of amputees should be educated on the burden of care associated with caregiving to amputees and the need to share the burden with the primary caregiver. Further, policymakers should consider making employment provisions for individuals who undergo amputation to ensure that amputees do not lose their jobs completely or are able to secure better alternatives that would accommodate their disability. Finally, both amputees and caregivers would benefit from education on the use of varied and adaptive coping strategies to enhance their well‐being.

## Author Contributions

**Esther Ohenewa:** conceptualisation, investigation, writing–original draft, methodology, writing–review and editing, formal analysis, project administration, data curation, resources. **Joana Salifu Yendork:** conceptualisation, investigation, writing–original draft, methodology, writing–review and editing, formal analysis, supervision. **Benjamin Amponsah:** conceptualisation, investigation, writing–original draft, methodology, writing–review and editing, formal analysis, supervision. **Frances Emily Owusu‐Ansah:** methodology, writing–review and editing, formal analysis, supervision.

## Ethics Statement

The researchers obtained ethical clearance from the Ethics Committee for Humanities (ECH: 016/16–17) at the University of Ghana, Legon, prior the commencement of the study.

## Conflicts of Interest

The authors declare no conflicts of interest.

## Data Availability

The data that support the findings of this study are available on request from the corresponding author.
